# Platelet Toll-Like Receptors Mediate Thromboinflammatory Responses in Patients With Essential Thrombocythemia

**DOI:** 10.3389/fimmu.2020.00705

**Published:** 2020-04-30

**Authors:** Cecilia P. Marín Oyarzún, Ana C. Glembotsky, Nora P. Goette, Paola R. Lev, Geraldine De Luca, María C. Baroni Pietto, Beatriz Moiraghi, Miguel A. Castro Ríos, Angeles Vicente, Rosana F. Marta, Mirta Schattner, Paula G. Heller

**Affiliations:** ^1^Institute of Medical Research A. Lanari, School of Medicine, University of Buenos Aires, Buenos Aires, Argentina; ^2^Department of Hematology Research, Institute of Medical Research (IDIM), National Scientific and Technical Research Council (CONICET), University of Buenos Aires, Buenos Aires, Argentina; ^3^Department of Hematology, Hospital General de Agudos José María Ramos Mejía, Buenos Aires, Argentina; ^4^Consultorios Hematológicos, Buenos Aires, Argentina; ^5^Department of Hematology, Hospital Alemán, Buenos Aires, Argentina; ^6^Laboratory of Experimental Thrombosis, Institute of Experimental Medicine (IMEX)- CONICET, National Academy of Medicine, Buenos Aires, Argentina

**Keywords:** essential thrombocythemia, platelet immunology, JAK2, thrombosis, inflammation, toll-like receptors

## Abstract

Essential thrombocythemia (ET) is comprised among chronic myeloproliferative neoplasms (MPN) and is caused by driver mutations in *JAK*2, *CALR*, and *MPL*, which lead to megakaryocyte proliferation and prominent thrombocytosis. Thrombosis remains the main cause of morbidity in ET and is driven by the interplay between blood cells, the endothelium, the clotting cascade, and host-derived inflammatory mediators. Platelet activation plays a key role in the thrombotic predisposition, although the underlying mechanisms remain poorly defined. In addition to their role in hemostasis, platelets participate in innate immunity and inflammation owing to the expression of toll-like receptors (TLR), which recognize inflammatory signals, triggering platelet functional responses. Considering the impact of inflammation on ET procoagulant state, we assessed the contribution of TLR2 and TLR4 to platelet hemostatic and inflammatory properties in ET patients, by using Pam3CSK4 and lipopolysaccharide (LPS) as specific TLR2 and TLR4 ligands, respectively. TLR2 ligation induced increased surface translocation of α-granule-derived P-selectin and CD40L, which mediate platelet interaction with leukocytes and endothelial cells, respectively, and higher levels of dense granule-derived CD63 in patients, whereas PAC-1 binding was not increased and LPS had no effect on these platelet responses. Platelet-neutrophil aggregate formation was elevated in ET at baseline and after stimulation of both TLR2 and TLR4. In addition, ET patients displayed higher TLR2- and TLR4-triggered platelet secretion of the chemokine RANTES (CCL5), whereas von Willebrand factor release was not enhanced, revealing a differential releasate pattern for α-granule-stored inflammatory molecules. TLR-mediated hyperresponsiveness contrasted with impaired or preserved responses to classic platelet hemostatic agonists, such as TRAP-6 and thrombin. TLR2 and TLR4 expression on the platelet surface was normal, whereas phosphorylation of downstream effector ERK1/2 was higher in patients at baseline and after incubation with Pam3CSK4, which may partly explain the enhanced TLR2 response. In conclusion, exacerbated response to TLR stimulation may promote platelet activation in ET, boosting platelet/leukocyte/endothelial interactions and secretion of inflammatory mediators, overall reinforcing the thromboinflammatory state. These findings highlight the role of platelets as inflammatory sentinels in MPN prothrombotic scenario and provide additional evidence for the close intertwining between thrombosis and inflammation in this setting.

## Introduction

Essential thrombocythemia (ET) is comprised among chronic myeloproliferative neoplasms (MPN) and is characterized by clonal proliferation of predominantly large hyperlobulated megakaryocytes in a normocellular bone marrow leading to sustained thrombocytosis. Driver mutations in *JAK2*, *CALR*, or *MPL* underlie most cases of ET, although the molecular pathogenesis of triple-negative cases remains largely undefined. Hyperactive JAK2 signaling is a common feature of MPN, irrespective of the type of driver mutation, and can be found even in triple-negative patients (1). Thrombosis remains the main cause of morbidity and mortality in ET and involves both arterial and venous thrombotic events. In addition, platelet aggregates formed under high shear stress conditions in the microvascular bed lead to the characteristic microcirculatory disturbances which are typically relieved by aspirin (2). The thrombotic predisposition in MPN results from the complex interplay among multiple factors (3). Those derived from the MPN clone comprise both excessive numbers and qualitative abnormalities in blood cells, which give rise to an activated prothrombotic phenotype that favors cellular interactions, endothelial activation and triggers the coagulation cascade (3, 4). In addition, inflammation has been shown to play a major role in MPN pathogenesis and, in this context, host-derived inflammatory cytokines impact on the MPN clone and further foster cellular activation, generating a self-reinforcing thromboinflammatory loop (4, 5). Activated platelets play a central role in ET prothrombotic state. Unequivocal evidence for *in vivo* platelet activation has been revealed by several studies and is reflected by platelet activation markers, including P-selectin and CD40L (6–8), platelet-leukocyte aggregates (6, 7) and elevated plasma levels of α-granule-derived molecules (2, 9). Factors leading to platelet activation remain incompletely defined. Both intrinsic platelet features induced by clonal mutations, cellular interaction with activated leukocytes and endothelial cells and hyperresponsiveness to soluble mediators have been proposed as likely mechanisms (4). Paradoxically, platelet activation may occur concomitantly with platelet dysfunction, which may be explained, at least in part, by *in vivo* degranulation of activated platelets and secondary storage pool deficiency (2).

In addition to their traditional function in hemostasis, recent evidence has revealed the key role of platelets in innate immunity and inflammation (10–12). Platelets participate in host defense owing to their ability to sense pathogens through the expression of functional toll-like receptors (TLRs), including plasma membrane-bound and endosomal receptors (13). Platelet surface TLRs include TLR4, which engages components of gram-negative bacteria, and TLR2, that recognizes gram-positive bacteria and may form heterodimers with either TLR1 or TLR6, whereas platelet endosomal receptors include TLR3, TLR7, and TLR9, which are mainly activated by viruses (13). Platelet TLR ligation elicits diverse proinflammatory as well as traditional prothrombotic activities of platelets (10, 13), providing a link between innate immunity and coagulation and contributing to pathological vascular occlusion in the setting of immunothrombosis. In particular, stimulation of TLR2/TLR1 by the synthetic lipopeptide Pam3CSK4 triggers platelet aggregation and granule secretion (14, 15), release of thromboinflammatory chemokines, such as RANTES (CCL5) and PF4 (CXCL4) (16), platelet-neutrophil aggregate formation and priming of platelet-induced neutrophil extracellular traps (NETs) (17). The effects of TLR2/TLR6 complex ligation depends on the nature of the ligand involved, as Mycoplasma-derived macrophage activating lipoprotein-2 (MALP-2) antagonizes TLR2/TLR1-mediated platelet responses (18), whereas the synthetic diacylated lipoprotein Pam2CSK4 triggers platelet activation (19). On the other hand, the direct effects of TLR4 ligation on platelet activation remain controversial. Whereas some groups reported that lipopolysaccharide (LPS)-induced TLR4 ligation induces direct platelet activation and/or granule release (20, 21) or potentiates the response to hemostatic agonists (15), others did not corroborate these findings (22). Nonetheless, it is well-established that LPS differentially modulates the release of α-granule-derived cytokines (23) and primes platelet-neutrophil aggregate formation (15) and platelet-mediated NET formation (17). In addition, LPS elicits platelet IL-1β RNA splicing and synthesis, providing further evidence for the involvement of TLR4 in platelet inflammatory responses (24).

Besides recognizing pathogens, TLR2 and TLR4 can also be stimulated by diverse endogenous ligands and thereby participate in thromboinflammatory reactions that take place in clinical conditions characterized by sterile inflammation, thus contributing to vascular disease (25). Previous findings from our group and others have revealed the presence of host-derived TLR ligands in MPN, including histone/DNA complexes (26), Hsp27 (27) and EDA-fibronectin (28), which engage TLR2 and/or TLR4. In order to determine the potential contribution of TLRs to platelet activation in ET, we assessed TLR2- and TLR4-mediated platelet thromboinflammatory responses, using the synthetic lipopeptide Pam3CSK4 and LPS, as prototypical TLR2/1 and TLR4 ligands, respectively, and employed classic hemostatic agonists, such as TRAP-6 and thrombin, for comparison.

## Patients and Methods

### Patients

Twenty patients with essential thrombocythemia (ET) diagnosed according to the 2016 WHO classification were included in this study. Clinical features are summarized in Table 1. Twenty healthy individuals were studied as controls and, in all cases, a control was studied simultaneously with each patient. Patients and controls were matched according to age, 53.3 (27–73) vs. 49 (26–76) years old, and sex, 70% and 65% were women, respectively. Platelet counts in controls were 223.5 (166–330) × 10^9^/L. Subjects were not taking aspirin nor drugs that may interfere with platelet function at the time of the study. This study was approved by the Ethics Committee of the Instituto de Investigaciones Médicas A. Lanari (protocol #241), University of Buenos Aires, and patients and controls gave written informed consent.

**TABLE 1:** Patient features at the time of the study.

	**Patients (*n* = 20)**
Age (years)	53.3 (27–73)
**Sex, *n* (%)**	
Female	13 (65)
Male	7 (35)
**Mutation, *n* (%)**	
*JAK2 V617F*	10 (50)
CALR*	7 (35)
Triple-negative	3 (15)
Platelet count (×10^9^/L)	679 (310–1715)
Hemoglobin (gr/dL)	13.55 (10–15.8)
Leukocyte count (×10^9^/L)	8.76 (3.9–11.5)
Prior thrombosis, *n* (%)**	3 (15)
Microvascular symptoms, *n*(%)	9 (45)
**Cytoreductive treatment, *n*(%)**	
None	16 (80)
Hydroxyurea	4 (20)
Time since diagnosis (months)	91.9 (0.4–365.7)

Values are reported as median and range. *All CALR + patients harbored type 1 mutations, except for one patient, who had type 2 mutation. **One patient had arterial thrombosis (AMI, stroke), one had venous thrombosis (portal vein thrombosis), and one had both arterial and venous thrombosis (stroke plus portal vein thrombosis). Two patients were on oral anticoagulants at the time of the study.

### Reagents

Lipopolysaccharide derived from Escherichia coli O111:B4, TRAP-6, ADP and prostaglandin (PG) E1 were obtained from Sigma-Aldrich (St. Louis, MO, United States). Pam3CSK4 was purchased from InvivoGen (San Diego, CA, United States) and thrombin from Biopool (Umea, Sweden). Fluorescein isothiocyanate (FITC)-conjugated mouse anti-human P-selectin (CD62P), anti-CD40L (CD154), and CD63 and PE-conjugated mouse anti-human CD41, TLR2, and TLR4 were from BD Biosciences (San Jose, CA, United States). FITC-conjugated CD45 and Human CCL5 (RANTES) ELISA Max were purchased from BioLegend (San Diego, CA, United States) and Quantikine ELISA Human P-selectin/CD62P was from R&D Systems (Minneapolis, MN, United States). Rabbit anti-human VWF and HRP-conjugated anti-human VWF were from Dako (Glostrup, Denmark), mouse anti- p-ERK1/2 (Tyr 204) and rabbit anti ERK 1/2 were obtained from Santa Cruz Biotechnology (Dallas, TX, United States).

### Platelet Activation by Flow Cytometry

Platelet-rich-plasma (PRP) was obtained from citrate-anticoagulated blood by centrifugation at 200 *g* during 10 min and adjusted to 100 × 10^9^/L with Tyrode’s buffer (134 mM NaCl, 12 mM NaHCO3, 2.9 mM KCl, 0.34 mM Na2HPO4, 1mM MgCl2, 1mM CaCl2, 10 mM Hepes, 5 mM glucose, pH 7.4). Platelets were stimulated with 10 ug/mL Pam3CSK4, 20 uM TRAP-6 or 20 uM ADP and labeled with FITC-conjugated anti-CD62P (P-selectin), anti-CD154 (CD40L), anti-CD63, PAC-1 or the corresponding IgG1 isotype controls for 15 min at 37°C. After stimulation, cells were fixed with 1% paraformaldehyde (PFA) and analyzed in a flow cytometer. The platelet population was identified by typical forward and side scatter features (Supplementary Figure S1A). Technical replicates are provided in Supplementary Table S1.

### Platelet-Neutrophil Aggregate Formation

To assess platelet-leukocyte aggregates, citrate-anticoagulated whole blood was adjusted to 60 × 10^9^/L leukocytes with phosphate-buffered saline and incubated with FITC-conjugated anti-CD45 and PE-conjugated anti-CD41 at basal state or after stimulation with 10 ug/mL Pam3CSK4, 10 ug/mL LPS or 20 uM TRAP-6 for 15 min at 37°C. Next, cells were fixed with 1% PFA and analyzed in a flow cytometer. The neutrophil population was selected according to CD45 expression and side scatter (Supplementary Figure S1B) and platelet-neutrophil aggregates were identified as the percentage of events staining positive for CD41. Technical replicates are provided in Supplementary Table S1.

### Levels of RANTES and von Willebrand Factor in the Platelet Releasate and Plasma by ELISA

Platelet-rich plasma was obtained from acid-citrate-dextrose (ACD)-anticoagulated blood in the presence of 1uM PGE1, washed twice with Tyrode’s buffer (pH = 6.5) and adjusted to 300 × 10^9^/L platelets in Tyrode’s buffer (pH = 7.4) to assess platelet secretion. Then, washed platelets were stimulated with 10 ug/mL Pam3CSK4, 10 ug/mL LPS, 0.5 IU/mL thrombin or 20 uM TRAP-6 during 30 min at 37°C. Activation was stopped with 1 uM PGE1, supernatants were collected after two centrifugation steps at 1100g and 9500g at 4°C, respectively, and preserved at -80°C until measured. Human CCL5 (RANTES) and von Willebrand factor (VWF) antigen were determined in the platelet releasate by ELISA using a commercial assay for RANTES and a homemade sandwich assay for VWF, as described (26). Plasma samples were obtained from EDTA-anticoagulated blood by two sequential centrifugation steps at 2500 *g* at 4°C and stored at −80°C until assayed. Plasma levels of RANTES and VWF were measured in plasma using the above-mentioned assays. In addition, soluble P-selectin levels were assessed in plasma by a commercial ELISA.

### Platelet Surface TLR 2 and TLR4 Expression

Platelet TLR2 and TLR4 expression was measured in EDTA-anticoagulated PRP adjusted to 60 × 10^9^/L platelets in the presence of PE-conjugated anti-TLR-2 or anti-TLR-4 for 30 min at room temperature. Cells were fixed with 1% PFA and analyzed by flow cytometry. Pam3CSK4-induced TLR2 expression was measured in citrate-anticoagulated PRP after incubation with 10 ug/mL Pam3CSK4 during 15 min at 37°C. Mean fluorescence intensity of TLR staining relative to the corresponding isotypic control was expressed as relative fluorescence intensity (RFI). Technical replicates are provided in Supplementary Table S1.

### Phosphorylation of ERK1/2 by Western Blot

Washed platelets (2 × 10^12^/L) were prepared as detailed above, incubated in resting conditions or stimulated with 10 ug/mL Pam3CSK4, 10 ug/mL LPS or 0.5 UI/mL thrombin for 15 min at 37°C. Lysates were prepared with RIPA buffer (50 mM Tris–HCl, pH 8, 150 mM NaCl, 1% Non-idet P-40, 0.1% SDS, 1% sodium deoxycholate) supplemented with protease and phosphatase inhibitors and 35 μg protein were resolved by SDS-PAGE. Immunoblotting was performed using mouse anti-phospho(p)-ERK1/2 (Tyr 204) and protein loading was assessed with rabbit anti-ERK1/2 followed by the corresponding HRP-conjugated secondary antibodies and detection by enhanced chemiluminescence. A ratio between pERK and ERK was calculated by densitometry.

### Statistical Analysis

Data were tested for Gaussian distribution and parametric or non-parametric tests were applied accordingly. For comparison between two groups, unpaired Student’s *t*-test or Mann-Whitney test were used according to data distribution. Correlations were analyzed by Spearman’s rank correlation coefficient. All statistical analyses were two-sided and *P* values <0.05 were considered significant. GraphPad Prism 7.02 software was used for statistical analysis.

## Results

### Platelet Activation Triggered by TLR Stimulation

**FIGURE 1:**
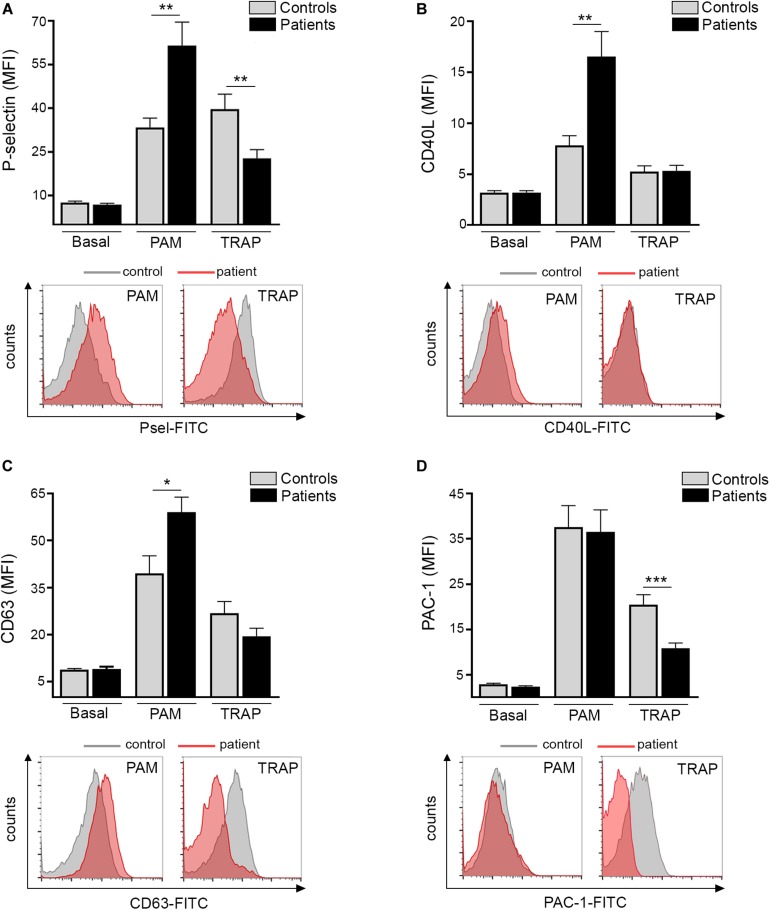
TLR-triggered platelet activation. Platelet-rich-plasma from patients (*n* = 20) and controls (*n* = 20) was stimulated with 10 μg/mL Pam3CSK4 (PAM) or 20 μM TRAP-6 and analyzed by flow cytometry for expression of cell adhesion molecules and activation markers. A healthy individual was studied in parallel with each patient. **(A)** Platelet surface P-selectin expression **(B)** CD40L translocation, **(C)** CD63 exposure, and **(D)** PAC-1 binding in resting, Pam3CSK4-, and TRAP-6- stimulated platelets. Data represent mean ± SEM mean fluorescence intensity (MFI). *P < 0.05, **P < 0.01, ***P < 0.001, unpaired *t*-test. Representative histograms for patient (red) and control (gray) platelets stimulated with Pam3CSK4 or TRAP-6 are shown for all parameters below each graph.

Platelet TLR2 ligation leads to surface translocation of α-granule adhesion molecules, such as P-selectin and CD40L, which mediate platelet interaction with leukocytes and endothelial cells, respectively (11, 12). Whereas baseline P-selectin did not differ between patients and controls, TLR2-agonist Pam3CSK4 triggered higher P-selectin levels in ET (Figure 1A). In striking contrast, P-selectin exposure induced by PAR-1 agonist, TRAP-6, was impaired (Figure 1A). Although baseline surface P-selectin was not increased in this cohort, evidence for *in vivo* platelet and/or endothelial activation was revealed by elevated soluble P-selectin in plasma (Supplementary Figure S2), suggesting P-selectin shedding. In accordance with P-selectin, Pam3CSK4-triggered exposure of CD40L was enhanced in patients, whereas no difference was found for TRAP-6 (Figure 1B). As shown for α-granule molecules, Pam3CSK4-triggered expression of dense granule and lysosome-derived CD63 was increased, with a trend toward decreased response to TRAP-6 (Figure 1C). Enhanced TLR2-mediated translocation of granule membrane proteins was not coupled to increase in a classic hemostatic response, such as GPIIbIIIa activation, as shown by normal Pam3CSK4-induced PAC-1 binding, whereas the response to TRAP-6 was impaired (Figure 1D). Decreased TRAP-6-induced P-selectin and PAC-1 was associated with reduced response to another hemostatic agonist, such as ADP (Supplementary Figure S3). In our system, no consistent induction of P-selectin, CD40L, CD63 and PAC-1 was achieved in platelets stimulated with LPS, in agreement with some but in contrast to other studies which evaluated the response of normal platelets to LPS (15, 20–22).

### Platelet-Neutrophil Interaction Primed by TLR Ligands

**FIGURE 2:**
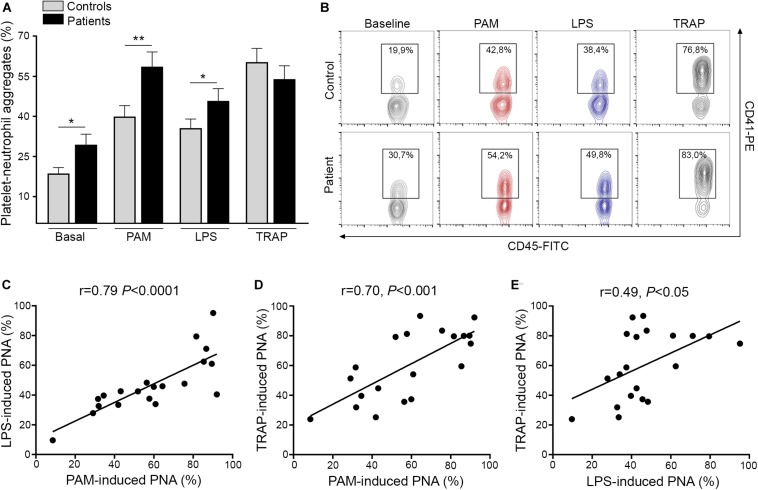
Platelet-neutrophil interaction induced by TLR ligation. **(A)** Platelet-neutrophil aggregate formation was assessed in whole blood from patients (*n* = 20) and controls (*n* = 20) at baseline and after stimulation with 10 μg/mL Pam3CSK4 (PAM), 10 μg/mL lipopolysaccharide (LPS), and 20 μM TRAP-6. Cells were stained with FITC-conjugated CD45 and PE-conjugated CD41 and analyzed by flow cytometry. The neutrophil population was selected according to CD45 expression and side scatter (SSC) and platelet-neutrophil aggregates were identified as the percentage of events staining positive for CD41. A healthy individual was studied in parallel with each patient. Data represent mean ± SEM values. *P < 0.05, **P < 0.01, unpaired *t*-test. **(B)** Representative images of platelet-neutrophil aggregates at baseline and triggered by Pam3CSK4, LPS, and TRAP-6. The percentage of CD45^+^CD41^+^ events is depicted in each graph. **(C–E)** Correlation between PAM-, LPS-, and TRAP-6-induced platelet-neutrophil aggregates (PNA) in essential thrombocythemia patients. Data were analyzed using Spearman correlation. Results are depicted in each graph.

Activated platelets engage neutrophils, promoting their reciprocal activation and functional responses (11, 12). In this cohort, we confirmed the presence of increased baseline circulating platelet-neutrophil aggregates in ET patients, as previously shown by us and other authors (6, 7, 26). Furthermore, both Pam3CSK4 and LPS triggered higher platelet-neutrophil complex formation in patients, whereas the response to TRAP-6 did not differ from controls (Figures 2A,B). Interestingly, tight correlation was shown in patients between Pam3CSK4- and LPS-mediated platelet-neutrophil aggregates and response to both immune agonists correlated also, although to a lesser degree, with the response to TRAP-6 (Figures 2C–E). In addition, although patients had elevated platelet counts, no correlation was found between platelet counts and platelet-neutrophil aggregates (Supplementary Figure S4), nor between platelet-neutrophil aggregates and P-selectin expression (Supplementary Figure S5). Overall, enhanced TLR-triggered platelet-neutrophil interaction could contribute to platelet and leukocyte activation in the MPN setting.

### TLR-Mediated Release of Platelet Thromboinflammatory Mediators

**FIGURE 3:**
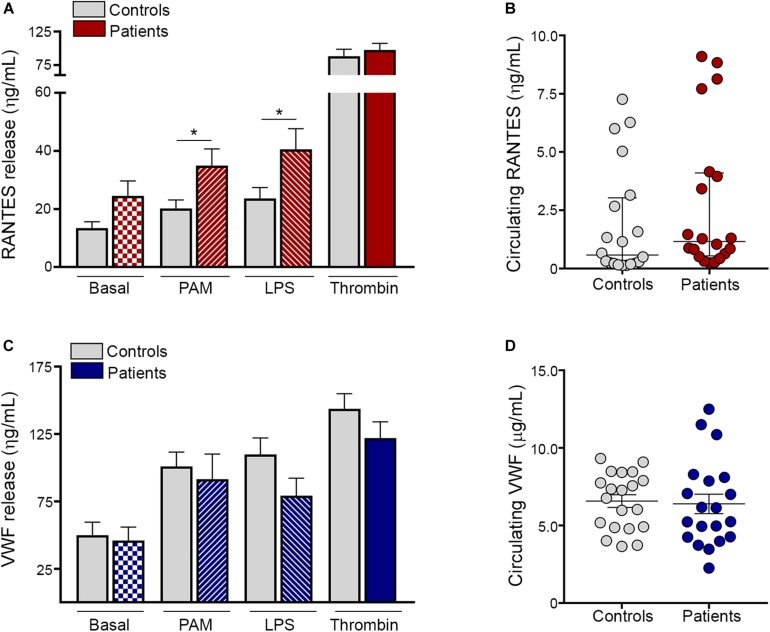
TLR-induced platelet release of proinflammatory mediators. Washed platelets from patients (*n* = 20) and controls (*n* = 20) were incubated in resting conditions or stimulated with 10 μg/mL Pam3CSK4 (PAM), 10 μg/mL lipopolysaccharide (LPS) or 0.5 U/L thrombin and the content of α-granule derived molecules was measured in the platelet supernatant by ELISA. **(A)** RANTES and **(C)** von Willebrand factor (VWF) levels in the platelet releasate. Values represent mean ± SEM. *P < 0.05, unpaired *t*-test. Circulating levels of **(B)** RANTES and **(D)** VWF in plasma from patients (*n* = 20) and controls (*n* = 20). Median values and interquartile range are depicted for RANTES and mean ± SEM values for VWF. *P* = NS, Mann-Whitney and Student’s *t*-test, respectively.

Besides expression of α-granule-derived adhesive molecules, activated platelets release a diverse array of α-granule-stored molecules, including the inflammatory chemokine RANTES, which is a potent chemoattractant for a variety of cells, including monocytes, and von Willebrand factor (VWF), which, in addition to its hemostatic function, plays an emerging role in vascular inflammation (12). We next assessed the secretion of these mediators by ET platelets. Baseline content of RANTES tended to be higher in the releasate of patient compared to control platelets. In addition, both Pam3CSK4 and LPS induced higher RANTES secretion in ET, whereas levels achieved after stimulation with a prototypic hemostatic agonist such as thrombin (Figure 3A) or TRAP-6 (Supplementary Figure S6A) did not differ from controls. As shown for platelet-neutrophil aggregates, a close correlation was found between the release of RANTES induced by Pam3CSK4 vs. LPS, while neither of them correlated with levels of RANTES achieved with hemostatic agonists (Supplementary Figure S7). Despite increased platelet RANTES release, levels of this chemokine were seldom elevated in patient circulation (Figure 3B). In contrast to RANTES secretion, baseline, Pam3CSK4- and LPS-triggered VWF release were not enhanced in ET and, similarly, no difference was found for thrombin (Figure 3C) or TRAP-6 (Supplementary Figure S6B). Plasma VWF levels were not significantly elevated in the overall patient cohort (Figure 3D) and no correlation was found between plasma and platelet-released VWF (data not shown), reflecting that endothelial cells rather than platelets represent the main source of VWF in circulation.

### Platelet TLR Expression

**FIGURE 4:**
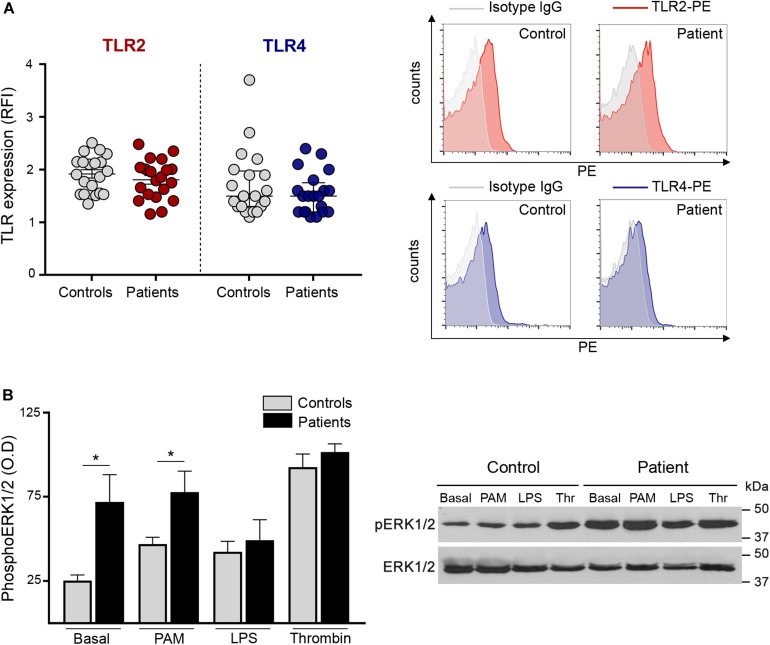
Platelet TLR expression and downstream signaling. **(A)** Surface expression of TLR2 and TLR4 was measured in patient (*n* = 20) and control (*n* = 20) platelets after incubation with PE-conjugated specific antibodies and the corresponding isotype IgG control. Cells were analyzed by flow cytometry and mean fluorescence intensity of TLR staining relative to isotype IgG was expressed as relative fluorescence intensity (RFI). Mean ± SEM for TLR2 and median and interquartile range for TLR4 are depicted. *P* = NS, Student’s *t*-test and Mann-Whitney test, respectively. Representative histograms of TLR2 (red) and TLR4 (blue) staining and the corresponding isotype controls (gray) are shown on the right panel for a patient and a control studied simultaneously. **(B)** Phosphorylation of ERK1/2 in patient (*n* = 8; 5 *JAK2*V617F^+^ and 3 *CALR*^+^) and control (*n* = 8) platelets. Washed platelets were incubated under resting conditions or after stimulation with 10 μg/mL Pam3CSK4 (PAM), 10 μg/mL lipopolysaccharide (LPS) and 0.5 U/L thrombin (Thr). Lysates were resolved by SDS-PAGE and immunoblotting was performed with mouse anti-phospho(p)ERK1/2. Protein loading was assessed with rabbit anti-ERK1/2 followed by the corresponding HRP-conjugated secondary antibodies and detection by enhanced chemiluminescence. The ratio between pERK1/2 and ERK1/2 was calculated by densitometry. *P < 0.05, unpaired *t*-test. A representative western blot image is shown on the right panel.

To assess whether the enhanced response to TLR agonists shown here by increased adhesion molecules, platelet-neutrophil aggregates and RANTES release, could be due to differences in receptor expression, we evaluated the levels of TLR2 and TLR4 on the platelet surface. Levels of these immune receptors did not differ between patients and controls (Figure 4A), excluding the possibility that receptor overexpression could account for TLR-induced hyperreactivity. Furthermore, TLR2 expression in platelets incubated with Pam3CSK4 was similar in patients and controls (Supplementary Figure S8). No substantial modulation of TLR4 expression was achieved with LPS (data not shown).

### TLR-Induced ERK1/2 Phosphorylation

Next, in an attempt to explain the differential response to inflammatory vs. hemostatic stimuli, we assessed intracellular signaling triggered by Pam3CSK4 and LPS in patient and control platelets and used thrombin for comparison. TLR stimulation results in the activation of several signaling pathways in platelets, including ERK1/2, PI3K/AKT, and NF-kB, all of which modulate TLR-triggered platelet responses (9, 14–16, 21). We focused on ERK1/2, which is essential to several platelet functional responses (29). Interestingly, baseline ERK phosphorylation was increased in patient platelets (Figures 4B,C). Although Pam3CSK4, LPS and thrombin triggered ERK phosphorylation in controls (*P* < 0.01, *P* < 0.05, and *P* < 0.0001, respectively), they failed to further increase ERK1/2 phosphorylation in patients. Nonetheless, pERK1/2 levels in platelets incubated with Pam3CSK4 remained higher in patients vs. controls, which may partly explain the enhanced TLR2-mediated responses, while, notably, LPS tended to attenuate pERK1/2 signal in patients, reaching levels similar to controls and thrombin-induced levels did not differ between both groups (Figures 4B,C).

### Relationship Between TLR-Mediated Platelet Responses and Clinical Features

Although JAK2-positive patients have been reported to have higher frequency of thrombosis and to display higher levels of several prothrombotic markers (3), no significant differences in TLR-triggered platelet responses were found between JAK2^+^ (*n* = 10) and CALR^+^ (*n* = 7) patients (Table 2). The vast majority of patients in this cohort were not receiving cytoreductive therapy. Interestingly, those receiving hydroxyurea (*n* = 4) had higher Pam3CSK4- and LPS-induced platelet RANTES secretion vs. those without treatment, although no differences were shown for other platelet responses (Supplementary Table S2). Of note, 3 of 4 HU-treated patients had had a previous thrombotic event, which occurred several years before inclusion in this study. On this regard, patients with previous thrombosis (*n* = 3) had higher levels of LPS-triggered platelet RANTES release compared to those without thrombosis and a similar trend was found for Pam3CSK4. The implications of higher RANTES release in patients with previous thrombosis or HU treatment are not clear and should be confirmed in a larger cohort. No differences in Pam3CSK4- and LPS-induced platelet parameters were evident between patients with (*n* = 9) or without microvascular disturbances (Supplementary Table S2).

**TABLE 2:** Comparison of TLR-triggered platelet responses between *JAK2*V617F^+^ and *CALR*^+^ patients.

	***JAK2 V617F*^+^ (*n* = 10)**	***CALR*^+^ (n = *7*)**	***P***
PAM-induced P-selectin (MFI)	56,6 ± 8,7	68,3 ± 18,8	NS
PAM-induced CD40L (MFI)	14,2 ± 3,3	20,4 ± 4,9	NS
PAM-induced CD63 (MFI)	65,5 ± 7,7	55,5 ± 7,3	NS
PAM-induced PAC-1 (MFI)	43,4 ± 6,6	33,1 ± 9,6	NS
PAM-induced PNA (%)	61,7 ± 6,9	69,5 ± 7,1	NS
LPS-induced PNA (%)	51,7 ± 6,5	49,6 ± 4,9	NS
PAM-induced RANTES release (ng/mL)	42,6 ± 10,1	28,6 ± 6,8	NS
LPS-induced RANTES release (ng/mL)	51,3 ± 12,9	33,1 ± 5,9	NS
PAM-induced VWF release (ng/mL)	94,6 ± 26,2	69,3 ± 35,9	NS
LPS-induced VWF release (ng/mL)	85,1 ± 20,5	60,8 ± 22,4	NS

*PAM means Pam3CSK4; LPS, lipopolysaccharide; MFI, mean fluorescence intensity; PNA, platelet-neutrophil aggregates. Mean ± SEM values are shown. *P* = NS (not significant), unpaired *t*-test or Mann-Whitney test.*

## Discussion

Platelet immune receptors play a key role in the complex intertwining of thrombosis and inflammation that takes place in the setting of several prothrombotic conditions associated with sterile inflammation, such as atherosclerosis, diabetes, cancer and autoimmune disorders (12). Emerging work highlights the fundamental contribution of systemic inflammation to MPN procoagulant state, as shown by the stepwise association of C-reactive protein levels and the thrombotic risk in both ET and Polycythemia Vera (5, 30). In this study, we show that upon ligand binding, activation of TLR2 and TLR4 leads to exacerbated platelet responses in ET patients, potentially contributing to thromboinflammation and vascular disease.

The MPN thromboinflammatory scenario involves multiple closely connected players, including activated platelets and leukocytes (3, 4). Platelet activation leads to surface translocation of α-granule-derived P-selectin which engages PSGL-1 on neutrophils and monocytes, initiating platelet-leukocyte crosstalk (12). Enhanced P-selectin exposure triggered by TLR2 activation shown in this study may contribute to exacerbated platelet-leukocyte interaction that takes place in ET, which has been shown to be critical to thrombosis development (3). In addition to P-selectin, ligation of TLR2 resulted in increased expression of another α-granule adhesion molecule, such as CD40L. Platelet CD40L favors platelet interaction with the endothelium via its CD40 counterreceptor leading to upregulation of endothelial adhesion molecules and proinflammatory cytokines (12). On this basis, increased TLR2-mediated platelet CD40L expression may contribute to platelet-endothelial interaction in ET. Together with α-granule-stored molecules, stimulation of TLR2 led to increased exposure of dense granule and lysosomal-derived CD63, whereas, in contrast to enhanced translocation of granular proteins, GPIIbIIIa activation, which represents a crucial step in platelet hemostatic function, was preserved. Considering that Pam3CSK4 selectively activates TLR2/TLR1, further work would be required to address whether TLR2-mediated hyperreactivity is limited to TLR2/TLR1 or involves other TLR2 partners, such as TLR2/TLR6. Strikingly, exacerbated TLR2/TLR1-mediated platelet activation contrasted with impaired response to classic hemostatic agonists, such as TRAP-6 and ADP. Although this work is, to our knowledge, the first to evaluate platelet responses to inflammatory mediators in ET, contradictory results have been reported regarding platelet activation triggered by classic hemostatic agonists. Whereas increased thrombin-induced P-selectin and preserved ADP response were shown in one study (6), impaired ADP- and TRAP-6-triggered P-selectin, CD63 and/or PAC-1 were shown in two other, coupled with intrinsic dysfunction of the PI3K/AKT pathway (31, 32). The finding of hyperresponsiveness to an immune stimulus (Pam3CSK4) vs. decreased response to hemostatic agonists (TRAP-6 and ADP) in this study indicates that the response of ET platelets may be influenced by the nature of the specific agonist involved, highlighting that inflammatory mediators may represent relevant drivers of platelet activation in this setting.

In addition to proinflammatory adhesion molecules, ligation of both TLR2 and TLR4 triggered increased platelet-neutrophil aggregate formation in ET patients, which could be due to the combined effect of TLR ligands on both platelets and neutrophils. No relationship was found between levels of these heterotypic complexes and P-selectin exposure, neither at baseline nor after Pam3CSK4 or TRAP-6 stimulation, probably reflecting that, besides P-selectin/PSGL-1, other molecular partners, such as Mac-1/GPIbα or GPIIbIIIa (via fibrinogen), mediate stable platelet/neutrophil interplay (12). Platelet-neutrophil crosstalk amplifies the activated state of both cell types and primes neutrophil function, including production of reactive oxygen species, which has been shown to be increased in MPN (26), and the release of neutrophil extracellular traps (NETs), whose role in MPN remains controversial (26, 33, 34). Collectively, enhanced TLR-triggered platelet-neutrophil interaction shown here may amplify both platelet and neutrophil activation and functional responses in ET.

A large scope of platelet activities, including those involved in inflammation and immunity, are mediated by the release of bioactive molecules stored in α-granules, which involve growth factors, angiogenesis mediators, hemostatic factors, and platelet-derived chemokines, such as RANTES (11, 12). In this study, baseline release of RANTES tended to be higher in patient compared to control platelets, and, moreover, both TLR2- and TLR4-mediated RANTES secretion were increased in ET. RANTES orchestrates several thromboinflammatory responses, including leukocyte chemoattraction and monocyte recruitment to the vessel wall, which is a critical step in atherogenesis (35). Furthermore, RANTES cooperation with PF4, which represents another platelet-derived chemokine, primes platelet-induced NET formation (36). In light of its functional effects, platelet delivery of RANTES and its deposition on inflamed or atherosclerotic endothelium may contribute to vascular disease in MPN, pointing to a role for platelets as a local source of inflammatory mediators in this scenario. Circulating RANTES derives from multiple cellular sources, being mainly released by T cells, although platelets and monocytes represent additional relevant sources. Despite higher platelet RANTES secretion, plasma levels of RANTES were seldom elevated in this patient cohort, indicating that RANTES may mediate local, but not systemic inflammation in ET. In contrast to platelet RANTES secretion, release of VWF was not enhanced in patients. The fact that RANTES release was not coupled to a similar pattern for VWF is intriguing. One potential explanation may involve differential α-granule secretion, as shown for molecules stored in distinct granule subpopulations, which are selectively released according to the triggering stimulus (37). As shown for platelet adhesion molecules, increased platelet-neutrophil aggregate formation and RANTES release triggered by TLRs was not coupled to enhanced response to platelet classic hemostatic agonists, reinforcing the finding of a differential behavior upon stimulation with immune vs. prothrombotic agonists. This selective TLR-mediated hyperresponsiveness was not due to TLR overexpression on the platelet surface, as no difference in TLR2 and TLR4 levels was found between patients and controls. Considering that previous data show that immune vs. thrombotic mediators induce differential activation of signaling cascades, including ERK1/2, in normal platelets, coupled to differences in platelet responses (38), we assessed whether differences in downstream signals may underlie the differential response to inflammatory vs. hemostatic stimuli shown by ET platelets. To this end, we focused on ERK1/2, which is essential in platelet activation and represents a JAK2-downstream effector shown to be hyperactivated in JAK2V617F-mutant cell lines (39) and MPN progenitors (40). Interestingly, ERK1/2 phosphorylation was increased in resting platelets from both JAK2-positive and CALR-positive patients, suggesting that, as shown for MPN nucleated cells, ERK1/2 is constitutively activated in ET platelets. Incubation with either immune or hemostatic agonists failed to trigger further ERK1/2 phosphorylation in patients, suggesting that ET platelets display maximal activation at baseline and are unable to respond to further stimulation. Nevertheless, ERK1/2 phosphorylation levels remained higher in patient vs. control Pam3CSK4-stimulated platelets, which may partly explain the enhanced response to TLR2 ligation shown in this work, while similar levels were reached with thrombin. Notably, LPS tended to attenuate ERK1/2 phosphorylation when compared to baseline in patients, suggesting dephosphorylation events could occur under this condition, as described for other cell types (41). Time-course experiments could be useful to further define this issue but are hampered by limited patient samples. In addition, study of other platelet signaling pathways may help to gain further insight into the mechanisms underlying the selective hyperresponsiveness to TLR ligation.

## Conclusion

**FIGURE 5:**
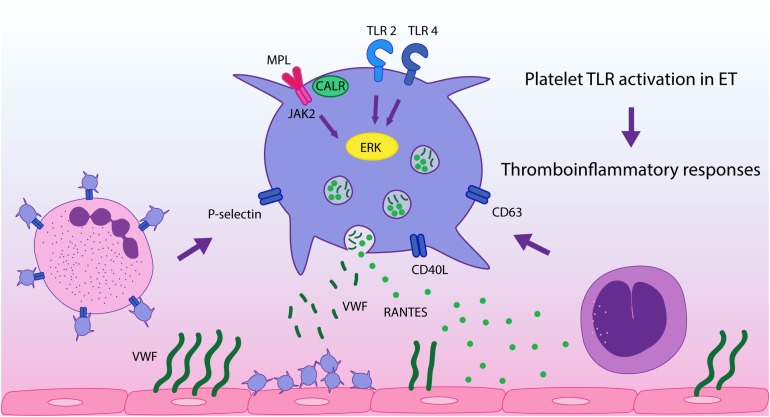
TLR-mediated platelet responses in essential thrombocythemia (ET). TLR2 stimulation leads to enhanced translocation of α-granule molecules P-selectin and CD40L, which mediate platelet interaction with leukocytes and the endothelium, respectively, and of dense granule-derived CD63. Activation of TLR2 and TLR4 in patient platelets triggers higher levels of platelet-neutrophil aggregates and higher release of RANTES, which is involved in monocyte chemoattraction to the vascular wall, while secretion of VWF is preserved. TLR and JAK2 activation converge on ERK1/2 signaling, which is hyperactivated in ET, contributing to TLR-hyperresponsiveness. These functional responses may reinforce ET thromboinflammatory state.

In conclusion, in this study we demonstrate that stimulation of platelet immune receptors, TLR2 and TLR4, leads to exacerbated thromboinflammatory responses in ET platelets and reveal a differential response pattern to inflammatory vs. hemostatic agonists. This finding, coupled to the presence of endogenous TLR ligands at steady state in MPN (26–28), provides an additional mechanism that may drive platelet activation in this context, promoting platelet-leukocyte and platelet-endothelial interaction and secretion of inflammatory mediators, as depicted in Figure 5. This phenomenon could be exacerbated during acute conditions, such as infections or tissue damage, which may trigger higher levels of TLR ligands. Our study emphasizes the role of platelets as inflammatory sentinels and key players in MPN prothrombotic scenario, highlighting both their hemostatic and immune properties, and provide additional evidence for the intertwining between thrombosis and inflammation in this setting. Further assessment of other TLRs relevant to platelet activation, such as endosomal TLR3, TLR7, and TLR9, could provide further insight into the inflammatory function of platelets in MPN.

## Data Availability Statement

The datasets generated for this study are available on request to the corresponding author.

## Ethics Statement

The studies involving human participants were reviewed and approved by the Comité de Etica del Instituto de Investigaciones Medicas Alfredo Lanari, Universidad de Buenos Aires. The patients/participants provided their written informed consent to participate in this study.

## Author Contributions

CM designed and performed the experiments, analyzed and interpreted the data, discussed the results, contributed to the manuscript draft, and prepared the figures. AG, NG, PL, GD, MB, and RM performed the experiments and discussed the results. MC, BM, AV, and PH provided the patient samples and clinical data. MS contributed to study design and discussed the results. PH designed and supervised the study, interpreted the data, and wrote the manuscript. All authors contributed to the editing and final approval of the manuscript.

## Conflict of Interest

The authors declare that the research was conducted in the absence of any commercial or financial relationships that could be construed as a potential conflict of interest.
