# Supplementary material for: Platelet Toll-Like Receptors Mediate Thromboinflammatory Responses in Patients With Essential Thrombocythemia
Source: Front Immunol. 2020 Apr 30;11:705. doi: 10.3389/fimmu.2020.00705 (PMC7203216; doi:10.3389/fimmu.2020.00705)
Supplement: Supplementary file 1 [file Data_Sheet_1.pdf]

**Supplementary Figures**

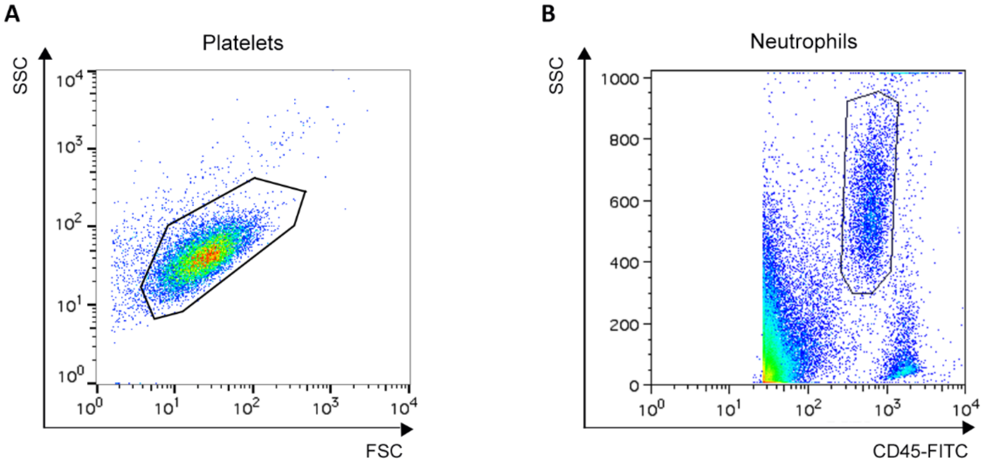


**Figure S1.** (A) Representative gating strategies for identifying the platelet population by forward (FSC) and side (SSC) scatter properties in platelet-rich plasma. (B) Representative gating strategies for identifying neutrophil population by CD45-FITC expression and SSC in whole blood to assess platelet-neutrophil aggregates.

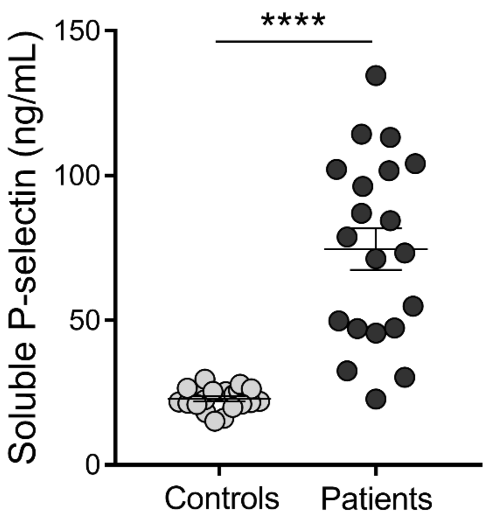


**Figure S2. Soluble P-selectin levels.** P-selectin was measured in plasma from patients (n=20) and controls (n=20) by ELISA. Mean ± SEM values are shown. **** *P*<0.0001, unpaired t-test.

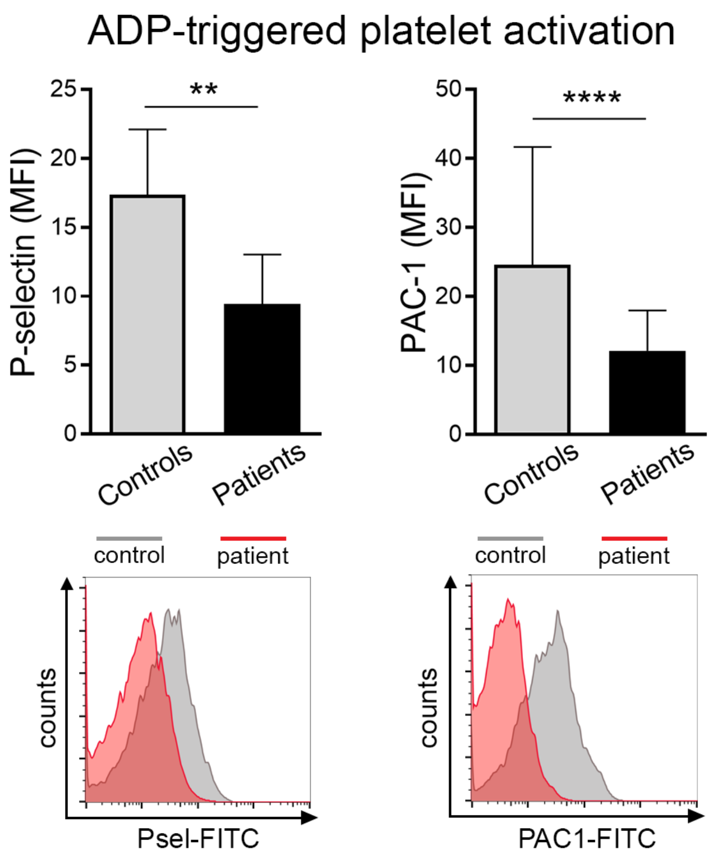


**Figure S3. ADP-triggered P-selectin exposure and PAC-1 binding**. Platelet-rich-plasma from patients (n=20) and controls (n=20) was stimulated with 20µM ADP and analysed by flow cytometry for (A) Surface P-selectin expression (B) PAC-1 binding. Bars represent median and interquartile mean fluorescence intensity (MFI). **$P$<0.01, ****$P$<0.0001, Mann-Whitney test. Representative histograms for patient (red) and control (gray) platelets stimulated with ADP are shown for each parameter.

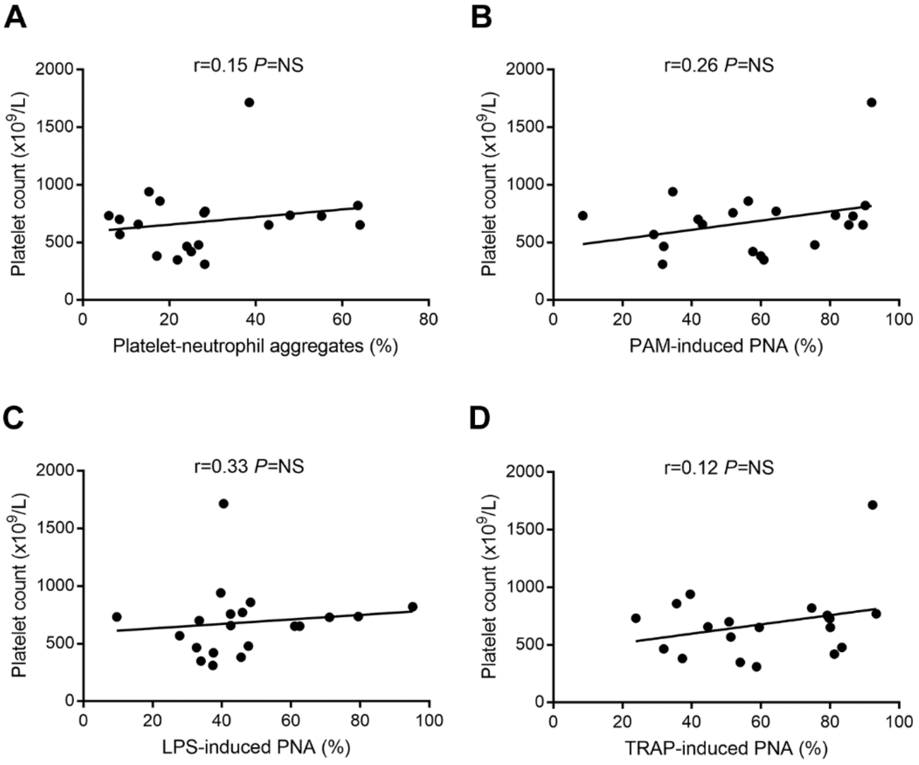


**Figure S4. Correlation between platelet counts and platelet-neutrophil aggregates.** Relationship between platelet-neutrophil aggregates (PNA) at (A) baseline or triggered by (B) Pam3CSK4 (PAM), (C) LPS, D) TRAP-6 vs. platelet counts in patients with essential thrombocythemia. Results are detailed for each graph. *P*=NS (not significant), Spearman correlation.

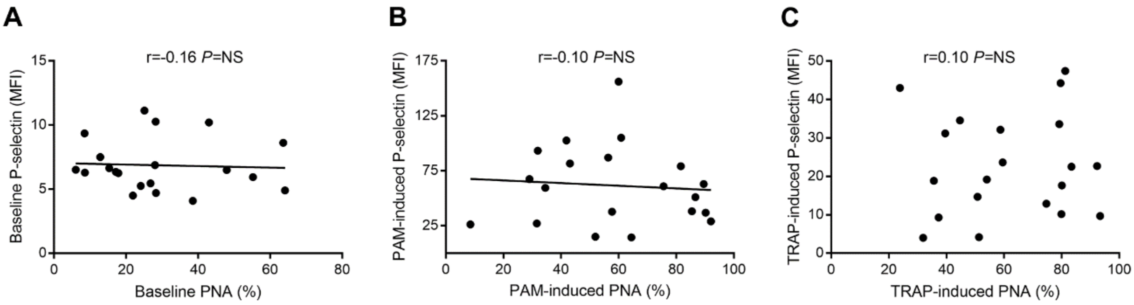


**Figure S5. Correlation between P-selectin and platelet-neutrophil aggregates.** Relationship between platelet-neutrophil aggregates (PNA) vs. platelet P-selectin expression at (A) baseline or triggered by (B) Pam3CSK4 (PAM) or TRAP-6 in patients with essential thrombocythemia. MFI means mean fluorescence intensity. Results are detailed for each graph. *P*=NS (not significant), Spearman correlation.

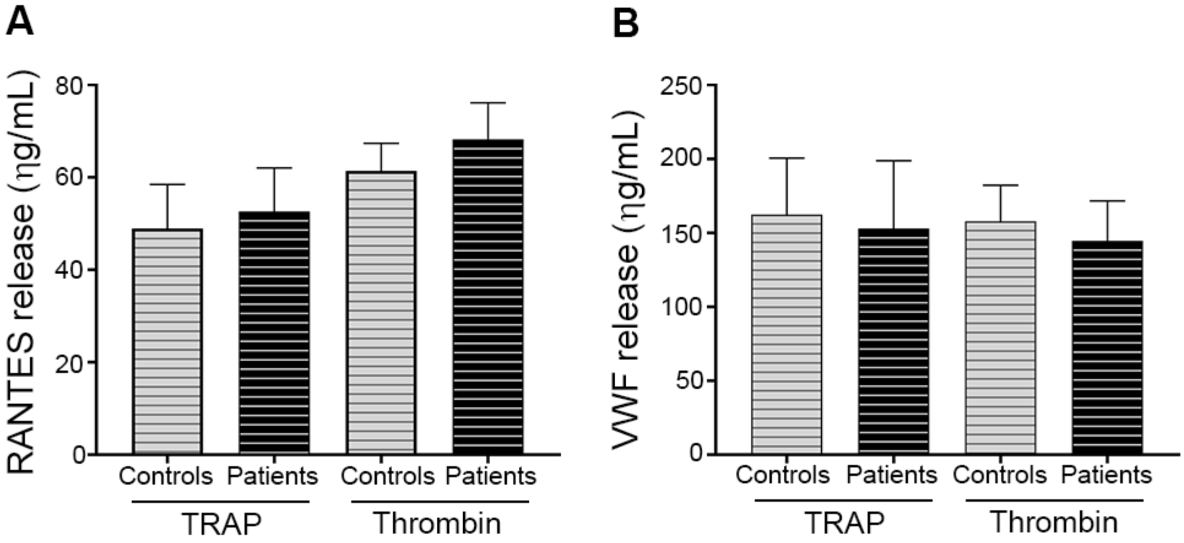


**Figure S6. Content of RANTES and von Willebrand factor (VWF) in the platelet releasate from TRAP-6-and thrombin-stimulated platelets.** Washed platelets from patients (n=6) and controls (n=6) were incubated in resting conditions or stimulated with 20µM TRAP-6 or 0.5 U/L thrombin and the content of α-granule derived molecules (A) RANTES and (B) VWF was measured in the platelet supernatant by ELISA. Values represent mean ± SEM. *P*=NS (not significant), unpaired t-test.

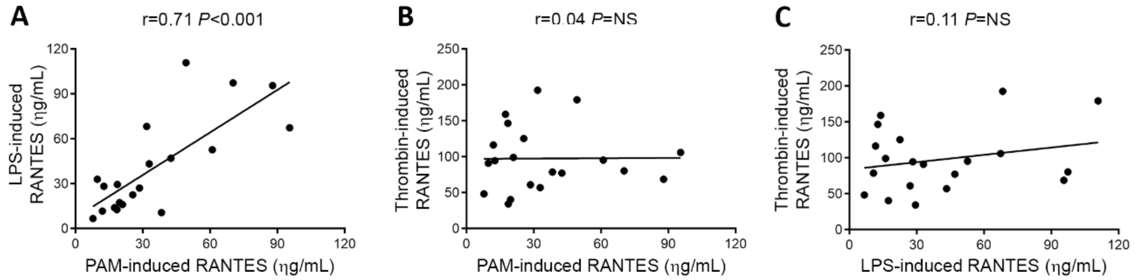


**Figure S7. Correlation between RANTES content in the platelet releasate induced by immune and hemostatic agonists.** Correlation between RANTES secretion triggered by (A) Pam3CSK4 (PAM) and LPS, (B) PAM and thrombin and (C) LPS and thrombin in platelets from essential thrombocythemia patients. Data were analysed by Spearman correlation and results are detailed above each graph.

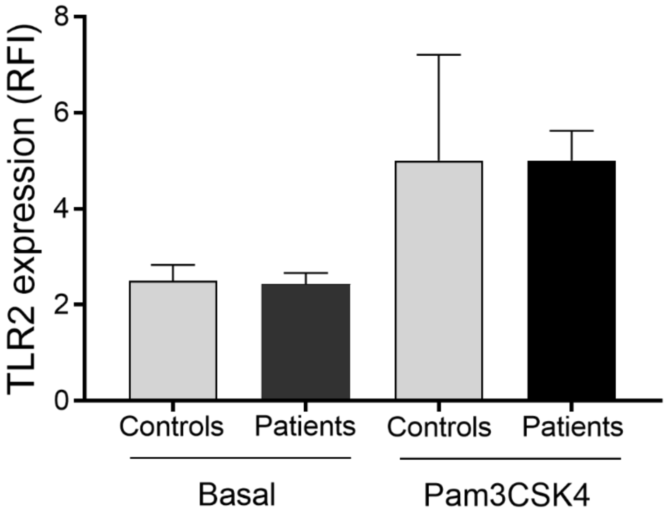


**Figure S8. TLR2 expression triggered by Pam3CSK4**. Surface expression of TLR2 was measured in patient (n=3) and control (n=3) platelets at baseline and after stimulation with 10µg/mL Pam3CSK4 (PAM). Cells were analysed by flow cytometry and mean fluorescence intensity of TLR2 staining relative to isotype IgG was expressed as relative fluorescence intensity (RFI). Median and interquartile range is depicted. *P*=NS, Mann-Whitney test.

Supplementary Tables

| | Mean ± SD |
|---|---|
| Basal P-selectin (MFI) | 1 ± 0.05 |
| PAM-induced P-selectin (MFI) | 1 ± 0.03 |
| TRAP-induced P-selectin (MFI) | 1 ± 0.11 |
| ADP-induced P-selectin (MFI) | 1 ± 0.01 |
| Basal CD40L (MFI) | 1 ± 0.09 |
| PAM-induced CD40L (MFI) | 1 ± 0.09 |
| TRAP-induced CD40L (MFI) | 1 ± 0.01 |
| Basal CD63 (MFI) | 1 ± 0.07 |
| PAM-induced CD63 (MFI) | 1 ± 0.03 |
| TRAP-induced CD63 (MFI) | 1 ± 0.04 |
| Basal PAC-1 (MFI) | 1 ± 0.03 |
| PAM-induced PAC-1 (MFI) | 1 ± 0.09 |
| TRAP-induced PAC-1 (MFI) | 1 ± 0.04 |
| ADP-induced PAC-1 (MFI) | 1 ± 0.06 |
| Basal PNA (%) | 1 ± 0.06 |
| PAM-induced PNA (%) | 1 ± 0.03 |
| LPS-induced PNA (%) | 1 ± 0.05 |
| TRAP-induced PNA (%) | 1 ± 0.02 |
| Surface TLR2 (RFI) | 1 ± 0.03 |
| Surface TLR4 (RFI) | 1 ± 0.05 |

**Table S1. Technical replicates for flow cytometry assays.** Mean ± standard deviation values are provided for each assay performed in triplicate with the same sample from a healthy individual and expressed relative to 1.

PAM means Pam3CSK4, LPS, lipopolysaccharide; PNA, platelet-neutrophil aggregates, MFI, mean fluorescence intensity; RFI, relative fluorescence intensity.

| | HU-treated n=4 | | Untreated n=16 | | *P* | Thrombosis n=3 | | w/o thrombosis n=17 | | *P* | MV n=9 | | w/o MV n=11 | | *P* |
|---|---|---|---|---|---|---|---|---|---|---|---|---|---|---|---|
| PAM-induced P-selectin (MFI) | 81,5 | ± 30,3 | 56,5 | ± 7,0 | NS | 56,6 | ± 24,4 | 62,4 | ± 8,8 | NS | 45,5 | ± 9,8 | 74,6 | ± 11,1 | NS |
| PAM-induced CD40L (MFI) | 16,5 | ± 8,0 | 16,5 | ± 2,5 | NS | 9,6 | ± 5,7 | 17,7 | ± 2,7 | NS | 12,7 | ± 3,7 | 19,7 | ± 3,1 | NS |
| PAM-induced CD63 (MFI) | 41,0 | ± 10,4 | 63,5 | ± 5,0 | NS | 43,6 | ± 14,2 | 61,7 | ± 5,0 | NS | 66,7 | ± 7,7 | 52,7 | ± 5,8 | NS |
| PAM-induced PAC-1 (MFI) | 48,6 | ± 11,2 | 33,5 | ± 5,4 | NS | 52,5 | ± 14,8 | 33,7 | ± 5,0 | NS | 32,9 | ± 6,6 | 39,4 | ± 7,2 | NS |
| PAM-induced PNA (%) | 52,5 | ± 7,0 | 60,2 | ± 6,7 | NS | 50,1 | ± 9,3 | 60,1 | ± 6,3 | NS | 63,1 | ± 9,4 | 55,0 | ± 6,6 | NS |
| LPS-induced PNA (%) | 38,6 | ± 2,5 | 48,8 | ± 5,3 | NS | 36,3 | ± 1,2 | 48,6 | ± 4,9 | NS | 46,3 | ± 7,6 | 47,1 | ± 5,1 | NS |
| PAM-induced RANTES (ηg/mL) | 57,6 | ± 15,5 | 29,4 | ± 5,4 | * | 65,8 | ± 18,5 | 29,6 | ± 5,1 | NS | 24,9 | ± 4,7 | 43,3 | ± 9,1 | NS |
| LPS-induced RANTES (ηg/mL) | 69,1 | ± 11,1 | 33,4 | ± 7,6 | * | 77,7 | ± 9,8 | 34,0 | ± 7,1 | * | 38,3 | ± 11,0 | 42,4 | ± 9,7 | NS |
| PAM-induced VWF (ηg/mL) | 119,1 | ± 65,0 | 84,7 | ± 17,9 | NS | 156,2 | ± 75,5 | 80,2 | ± 17,4 | NS | 96,1 | ± 25,1 | 87,9 | ± 27,9 | NS |
| LPS-induced VWF (ηg/mL) | 103,8 | ± 33,8 | 73,1 | ± 14,2 | NS | 124,7 | ± 37,5 | 71,2 | ± 13,4 | NS | 77,4 | ± 21,9 | 80,7 | ± 16,6 | NS |

**Table S2. Comparison of TLR-triggered platelet responses in essential thrombocythemia patients according to clinical features.**

HU means hydroxyurea; w/o, without; MV, microvascular circulatory disturbances; PAM, Pam3CSK4; LPS, lipopolysaccharide; MFI, mean fluorescence intensity; PNA, platelet-neutrophil aggregates.

Mean ± SEM values are shown. *$P$<0.05; $P$= NS (not significant), unpaired t-test or Mann-Whitney test.
